# *Alu* Exonization Events Reveal Features Required for Precise Recognition of Exons by the Splicing Machinery

**DOI:** 10.1371/journal.pcbi.1000300

**Published:** 2009-03-06

**Authors:** Schraga Schwartz, Nurit Gal-Mark, Nir Kfir, Ram Oren, Eddo Kim, Gil Ast

**Affiliations:** Department of Human Molecular Genetics and Biochemistry, Sackler Faculty of Medicine, Tel-Aviv University, Tel Aviv, Israel; Center for Genomic Regulation, Spain

## Abstract

Despite decades of research, the question of how the mRNA splicing machinery precisely identifies short exonic islands within the vast intronic oceans remains to a large extent obscure. In this study, we analyzed *Alu* exonization events, aiming to understand the requirements for correct selection of exons. Comparison of exonizing *Alu*s to their non-exonizing counterparts is informative because *Alu*s in these two groups have retained high sequence similarity but are perceived differently by the splicing machinery. We identified and characterized numerous features used by the splicing machinery to discriminate between *Alu* exons and their non-exonizing counterparts. Of these, the most novel is secondary structure: *Alu* exons in general and their 5′ splice sites (5′ss) in particular are characterized by decreased stability of local secondary structures with respect to their non-exonizing counterparts. We detected numerous further differences between *Alu* exons and their non-exonizing counterparts, among others in terms of exon–intron architecture and strength of splicing signals, enhancers, and silencers. Support vector machine analysis revealed that these features allow a high level of discrimination (AUC = 0.91) between exonizing and non-exonizing *Alu*s. Moreover, the computationally derived probabilities of exonization significantly correlated with the biological inclusion level of the *Alu* exons, and the model could also be extended to general datasets of constitutive and alternative exons. This indicates that the features detected and explored in this study provide the basis not only for precise exon selection but also for the fine-tuned regulation thereof, manifested in cases of alternative splicing.

## Author Summary

A typical human gene consists of 9 exons around 150 nucleotides in length, separated by introns that are ∼3,000 nucleotides long. The challenge of the splicing machinery is to precisely identify and ligate the exons, while removing the introns. We aimed to understand how the splicing machinery meets this momentous challenge, based on *Alu* exonization events. *Alus* are transposable elements, of which approximately one million copies exist in the human genome, a large portion of which within introns. Throughout evolution, some intronic *Alus* accumulated mutations and became recognized by the splicing machinery as exons, a process termed exonization. Such *Alus* remain highly similar to their non-exonizing counterparts but are perceived as different by the splicing machinery. By comparing exonizing *Alus* to their non-exonizing counterparts, we were able to identify numerous features in which they differ and which presumably lead to the recognition only of the former by the splicing machinery. Our findings reveal insights regarding the role of local RNA secondary structures, exon–intron architecture constraints, and splicing regulatory signals. We integrated these features in a computational model, which was able to successfully mimic the function of the splicing machinery and discriminate between true *Alu* exons and their intronic counterparts, highlighting the functional importance of these features.

## Introduction

How are short exons, embedded within vast intronic sequences, precisely recognized and processed by the splicing machinery? Despite decades of molecular and bioinformatic research, the features that allow recognition of exons remain poorly understood. Various factors are thought to be of importance. These include the splicing signals flanking the exon at both ends, known as the 5′ and 3′ splice sites (5′ss and 3′ss, respectively), auxiliary cis-elements known as exonic and intronic splicing enhancers and silencers (ESE/Ss and ISE/S) that promote or repress splice-site selection, respectively [1],[2], and exon [3] and intron length [4]. There is an increasing body of evidence that secondary structure is a powerful modifier of splicing events [5]–[12]. Secondary structure is thought to present binding sites for auxiliary splicing factors, correctly juxtapose widely separated cis-elements, and directly affect the accessibility of the splice sites. However, only very few studies have used bioinformatic approaches to broadly study the effects of secondary structure on splicing [13]–[15].

Many of the above-listed factors have been subjected to analysis in the context of comparison between constitutively and alternatively spliced exons. It has been found, for example, that constitutively spliced exons are flanked by stronger splicing signals, that they contain more ESEs but fewer ESSs, and are longer but flanked by shorter introns with respect to their alternatively spliced counterparts (reviewed in [16]). However, to what extent do these features contribute to the selection of exons and allow discrimination between true exons and “non-exons”, i.e. sequences resembling exons but not recognized by the splicing machinery? This question is fundamental for understanding the process of exon selection by the spliceosome, and yet has not been subjected to much analysis. This is presumably because unlike alternatively and constitutively spliced exons, both of which are relatively easy to define computationally, defining a non-exon or a pseudo-exon is more of a challenge. One approach is to compare exons to sequences of up to a certain length which are flanked by splicing signals exceeding a certain threshold [17],[18]. Although this approach is powerful and has contributed to the discovery of the “vocabulary” of exons, it is also limited. The primary limitation is that it is circular: For the mere definition of pseudo-exons, we are forced to fix various features—such as minimal splice site strength and exon length—that we would prefer to infer.

To circumvent these obstacles, we have studied *Alu* exonization events. *Alu* elements are primate-specific retroelements present at about 1.1 million copies in the human genome. A large portion of *Alu* elements reside within introns [19]. *Alu*s are dimeric, with two homologous but distinct monomers, termed left and right arms [20]–[22]. During evolution, some intronic *Alu*s accumulated mutations that led the splicing machinery to select them as internal exons, a process termed exonization [23]–[25]. Such exonization events may occur either from the right or the left arm of the *Alu* sequence, but are observed predominantly in the antisense orientation relative to the mRNA precursor. Almost invariably, such events give birth to an alternatively spliced exon, as a constitutively spliced exon would compromise the original transcriptomic repertoire and hence probably be deleterious [19],[24],[26],[27]. The fact that exonizing and non-exonizing *Alu*s have retained high sequence similarity but are perceived as different by the splicing machinery makes them excellent candidates for studying the factors required for precise recognition of exons by the spliceosome. The natural control group of non-exonizing *Alu*s obviates the need to fix different parameters in the control set, and the high degree of sequence similarity shared by all *Alu*s, regardless of whether they do or do not undergo exonization, enables direct comparison of a wide array of features.

Based on the comparison between *Alu* exons and their non-exonizing counterparts, we were able to identify several key features that characterize *Alu* exons and to determine the relative importance of these features in the process of *Alu* exonization. A novel result of this comparison was the importance of pre-mRNA secondary structure: More thermodynamically stable predicted secondary structure in an *Alu* arm harboring a potential *Alu* exon decreases the probability of an exonization event originating from this *Alu*. Thus, this study is among the first to provide wide-scale statistical proof of the importance of secondary structure in the context of exon selection. We identified numerous further factors differentiating between *Alu* exons and non-exons, and integrated them in a machine learning classification model. This model displayed a high performance in classifying *Alu* exons and non-exons. Moreover, the strength of predictions by this model correlated with biological inclusion levels, and higher probabilities of exonization were given by the model to constitutive exons than to alternative ones. These findings indicate that the features identified in this study may form the basis for precise exon selection, and make the difference between a non-selected element, an alternatively-selected element, and a constitutively selected one.

## Results

### Compilation of Datasets

**Figure 1:**
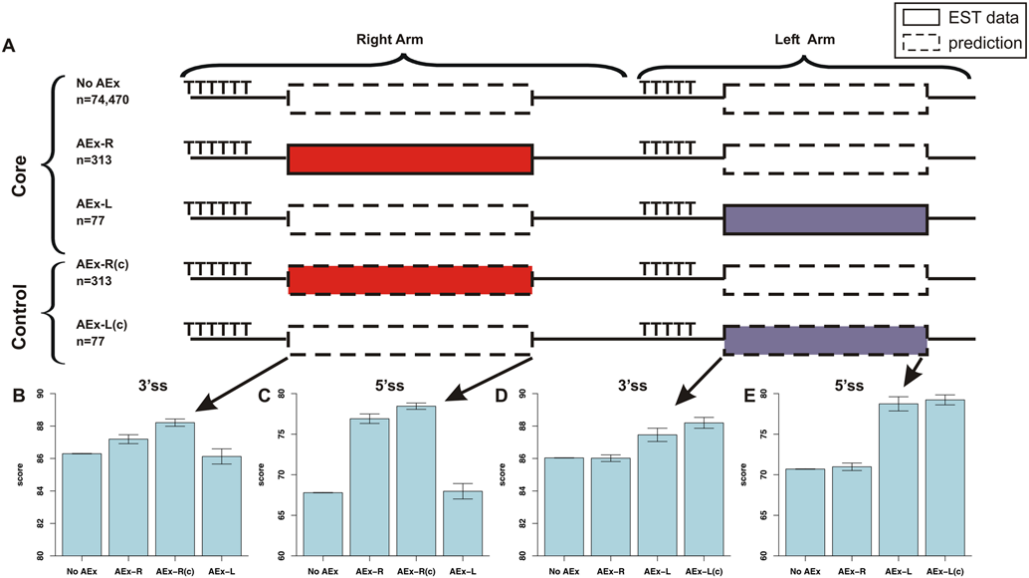
Diagram of the five datasets and splicing signal scores for each dataset. (A) Diagram depicting the three core datasets and the two control datasets used throughout the study. Each dataset is represented by an illustration of an *Alu* sequence in the antisense orientation. The right and left arms are indicated, along with the typical poly-T stretch located at the 5′ end of each arm. Boxes in the left and right arms mark the potentially exonizing sequence. Solid box borders mark exon boundaries derived based on EST data, whereas dashed borders mark exon boundaries based on bioinformatic predictions, as indicated by the legend on the upper right. The exons in the exonizing groups and in the corresponding control groups, were visualized in the same colors, to emphasize that the latter serves as a control to the former. (B–E) 3′ss and 5′ss scores in the two arms of the *Alu* element across the different core datasets, as well as for the relevant control dataset.

We set out to determine the features underlying the recognition of *Alu* exons by the splicing machinery. We therefore required datasets of *Alu*s that undergo and that do not undergo exonization. We took advantage of the fact that *Alu* elements may exonize either from the right or from the left arm, and composed three core datasets (Figure 1A): (1) A dataset of 313 *Alu* exons (AEx) that are exonized from the right *Alu* arm, termed AEx-R; (2) A dataset of 77 *Alu*s that undergo exonization in the left arm, termed AEx-L; (3) A dataset of 74,470 intronic *Alu*s lacking any evidence of exonization, called No AEx. In all these datasets, *Alu*s had to be embedded in the antisense orientation within genes, since most exonization events of *Alus* occur in this orientation [19],[23],[28]. Finally, to allow direct comparison between parallel positions in different *Alu*s, we used pairwise alignments to align each *Alu* in each of the datasets against an *Alu* consensus sequence.

We next computationally searched for the optimal borders, or splice sites, of non-exons within both the right arm and the left arm of the sequences in the No AEx dataset. This was done in two steps: (1) We first empirically determined the positional windows in which the selected 3′ss and 5′ss appeared within exonizing *Alus*; (2) We next searched the above-determined positional windows for the highest scoring splicing signals (see Materials and Methods). We found that computational selection of the highest scoring splicing signal yielded a high extent of congruence (ranging between 74%–96%, depending on the arm and on the signal) with the “true” splicing signal based on EST data. Since the congruence was not perfect, we created two control datasets based on the AEx-R and AEx-L group, termed AEx-R(c) and AEx-L(c), respectively, in which exon borders were searched for computationally as in the No AEx dataset. These two subsets were used to verify that differences between the exonizing and non-exonizing datasets were not due to the manner in which exons and non-exons were derived (ESTs versus computational predictions). To complete the picture, we computationally searched for non-exons within the right arm of the AEx-L group and in the left arm of the AEx-R group. Notably, we demanded that all exons within all datasets have a minimal potential 3′ss (AG) and 5′ss (GT/GC), because lacking such minimal conditions *Alus* cannot undergo exonization at all. Thus, our analyses are based on three core and two control sets of *Alus* with two sets of start and end coordinates mapped for each *Alu*—one in the right arm and one in the left (see Materials and Methods for further details).

### *Alu* Exons Are Flanked by Stronger Splicing Signals Than Their Non-Exonizing Counterparts

Previous studies, based on much smaller datasets, implicated the 3′ss [24] and the 5′ss [26] splicing signals as major factors determining exonization events. To assess whether this held for our dataset as well, we calculated the strength of the 5′ss and 3′ss of the exons/non-exons in the right and in the left arms in each of the five datasets. Indeed, we found that in the right arms the 3′ss and the 5′ss scores were highest among those *Alu*s that underwent exonization (Figure 1B and 1C, respectively). Similarly, in the left arms, the scores of the 3′ss and the 5′ss are highest among the exonizing *Alu*s (Figure 1D and 1 E, respectively). These results were highly statistically significant (see Text S1). Moreover, these differences are even more pronounced when comparing the two control datasets to their non-exonizing counterparts (compare the results for AEx-R and AEx-L to AEx-R(c) and AEx-L(c), respectively, in Figure 1B–E). Thus, these analyses fit in with previous analyses emphasizing the role of the two major splicing signals.

### Exonizing *Alu*s Have Less Stable Secondary Structures Than Their Non-Exonizing Counterparts

**Figure 2:**
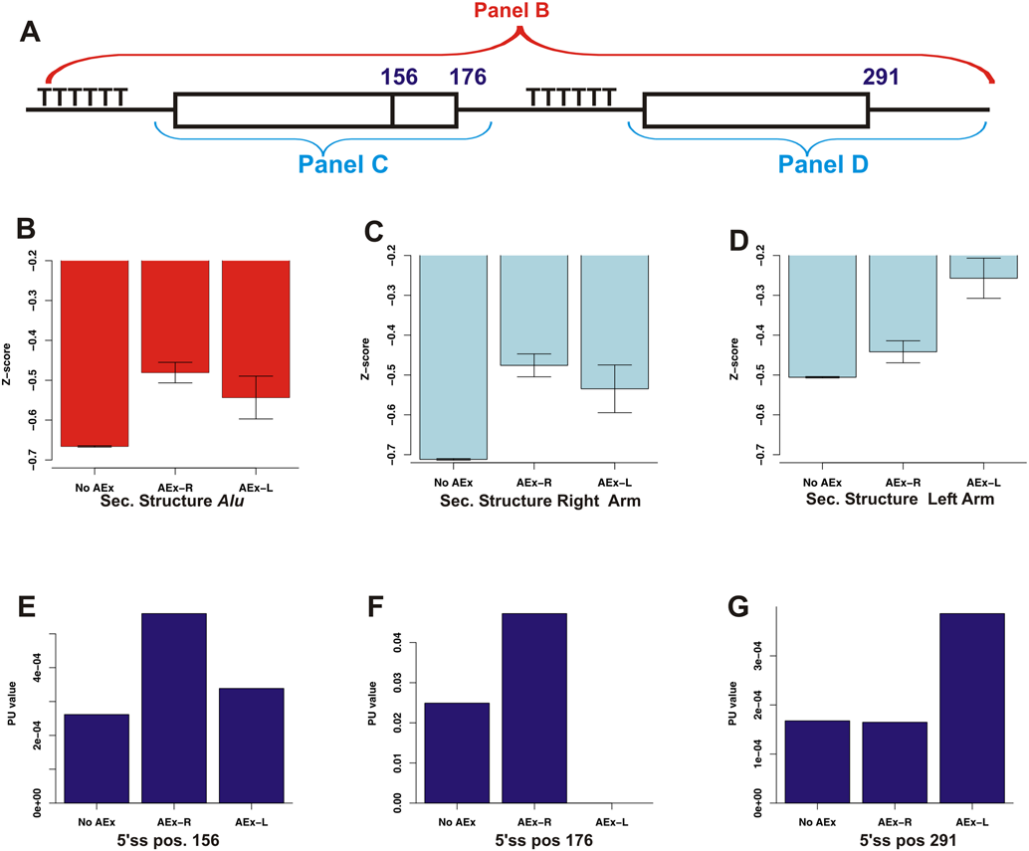
Impact of local secondary structure on exonization. (A) Diagram representing an *Alu* sequence, as in Figure 1A, indicating which parts of the *Alu* sequence were folded in (B–D). The positions of the three main 5′ splice sites, used in (E–G), are indicated as well. (B) Comparison of predicted secondary structure free energy upon folding of the entire *Alu* sequence, with lower Z-scores representing stronger secondary structures (see text). The mean Z-scores (y-axis) are shown for the *Alu*s in each of the three core datasets. Error bars mark the standard error of the means. (C) Comparison of the predicted secondary structure free energy upon folding of the right arm only. (D) Comparison of the predicted secondary structure free energy upon folding of the left arm only. (E–G) PU values in each of the three main positions serving as 5′ss across the three core datasets. Higher PU values indicate that the 5′ss is less likely to form part of a secondary structure.

We were interested in assessing the role of secondary structure in the context of *Alu* exonization events. We therefore began by computing the thermodynamic stabilities of the secondary structures predicted for the *Alu*s in each of the core datasets. We used RNAfold [29] to calculate the secondary structure partition function; but rather than use this metric directly, we used a dinucleotide randomization approach to yield a Z-score that is not sensitive to sequence length or nucleotide composition (see Materials and Methods). We found that *Alu*s that gave rise to exonization events, regardless of whether from the left or from the right arm, were characterized by weaker secondary structures than *Alu*s that do not undergo exonization (Figure 2A). This was highly significant in the case of exonizations originating from the right arm (AEx-R vs. No AEx p = 9.8E−12) and of borderline significance for the left arm exonizations (AEx-L vs. No AEx p = 0.07). This provided the first indication that strong secondary structures might prevent *Alu* exonizations.

To pinpoint the subsequences to which the differences in strength of secondary structure could be attributed, we next calculated secondary structure Z-scores for each of the two *Alu* arms separately. We found that the secondary structures of right and the left arms were weakest in cases in which these arms undergo exonization (Figure 2B and 2C, respectively). These changes relative to the No AEx group were highly significant (p = 2E−15 and p = 1.08E−5, respectively). Interestingly, the non-exonizing arm tended to have weaker secondary structure in those cases in which the opposite arm underwent exonization (p = 0.001 when comparing the left arm of the AEx-R to the No AEx dataset, and p = 0.055 when comparing the right arm of the AEx-L to the No AEx dataset). These observations suggested that secondary structures have a detrimental effect on the recognition of *Alu* exons primarily when the structure incorporates sequence from the exon itself, but also when stable structures are located in relative proximity to the exon.

### The 5′ss of *Alu* Exons Tends To Be Unstructured

Secondary structure has been shown to impair exon recognition by affecting the accessibility of splice sites [8],[9],[11],[12],[30]. To examine whether sequestration of splice sites within secondary structures plays a role in the context of *Alu* exonizations, we used a measure indicating the probability that all bases in a motif are unpaired (denoted probability unpaired or PU value) [31]. Briefly, this measure indicates the probability that a motif, located within a longer sequence, is participating in a secondary structure. Higher values indicate that the motif is more likely to be single stranded and lower values indicate a greater likelihood of participating in a secondary structure (see Materials and Methods). We assessed the single strandedness of the two most frequently selected 5′ss in the right arm located at positions 156 and 176 relative to the consensus (also termed sites B and C [28]) and the most frequently selected 5′ss of the left arm, located at position 291 (see Figure 2A). We found that 5′ss selected in exonization events are characterized by significantly higher PU values than their non-exonizing counterparts, indicating that selected 5′ss have a lower tendency to participate in secondary structures (see Figure 2E–G). We repeated this analysis for the two most frequently selected 3′ss in the right arm and the most frequently selected 3′ss in the left arm, but did not observe higher single-strandedness in the selected 3′ss with respect to their non-selected counterparts (data not shown). However, this finding may also be attributed to the fact that all *Alus*, regardless of whether they undergo exonization or not, are characterized by relatively strong 3′ss, due to the poly-T stretch characterizing them (see Discussion). See Text S1 for description of a control analysis.

### Exons Are 10 nt Longer But Flanked by Dramatically Shorter Introns

**Figure 3:**
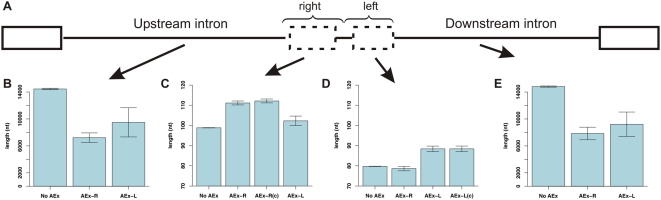
Analysis of lengths of the *Alu* exons/non-exons and flanking introns. (A) Diagram depicting the exon-intron architecture of a representative *Alu*. The potential sites of exonization are denoted by dashed boxes, the lines flanking these sites represent introns, and the exons flanking the *Alu* exon/non-exon are marked by solid boxes. (B,E) Lengths of introns upstream and downstream of the *Alu* sequence, respectively, in the groups No AEx, AEx-R, and AEx-L. (C,D) Exon/non-exon lengths; data is presented for the three core groups, as well as for the relevant control group (either AEx-R(c) or AEx-L(c)).

Intron-exon architecture has well-documented effects on splicing. Therefore, we compared the lengths of the *Alu* exons to their counterpart non-exons (diagram in Figure 3A). We found that exons were ∼10 nt longer than their non-exonizing counterparts (Figure 3C and 3D). Exons in the right arm of the AEx-R dataset were 112 nt long, on average, whereas non-exons were only 102 nt long in the No AEx dataset. The same trend was observed in the AEx-L dataset: Exons in the left arm of the AEx-L dataset were 88 nt long, whereas the non-exons in the No AEx group were 78 nt long. In both cases, the differences were highly statistically significant (see Text S1). This indicates that increased exon length is an advantage in terms of exonization of *Alu* elements.

Analyzing the lengths of the flanking introns, we found that introns flanking *Alu* exons were almost 50% shorter than those flanking their non-exonizing counterparts. Introns upstream of *Alu* exons in the AEx-R or AEx-L dataset were 7,216 and 9,497 nt long, respectively, on average (Figure 3B), but 14,458 nt long upstream of the non-exons in the No AEx group. These differences were highly significant (No AEx vs. AEx-R p = 1.38E−13, No AEx vs. AEx-L p = 0.0047). Highly significant findings were observed in the downstream intron as well. These introns were 7,844 and 9,210 nt long for exons in the AEx-R and AEx-L dataset, respectively, but 14,808 nt long for *Alu*s in the No AEx dataset (Figure 3E). Taken together, these results indicate that recognition of exons by the splicing machinery correlates positively with exon length but negatively with intron length, yielding insight on the constraints and the mechanism of the splicing machinery (see Discussion).

### *Alu* Exons Are Enriched in Enhancers and Depleted in Silencers

**Figure 4:**
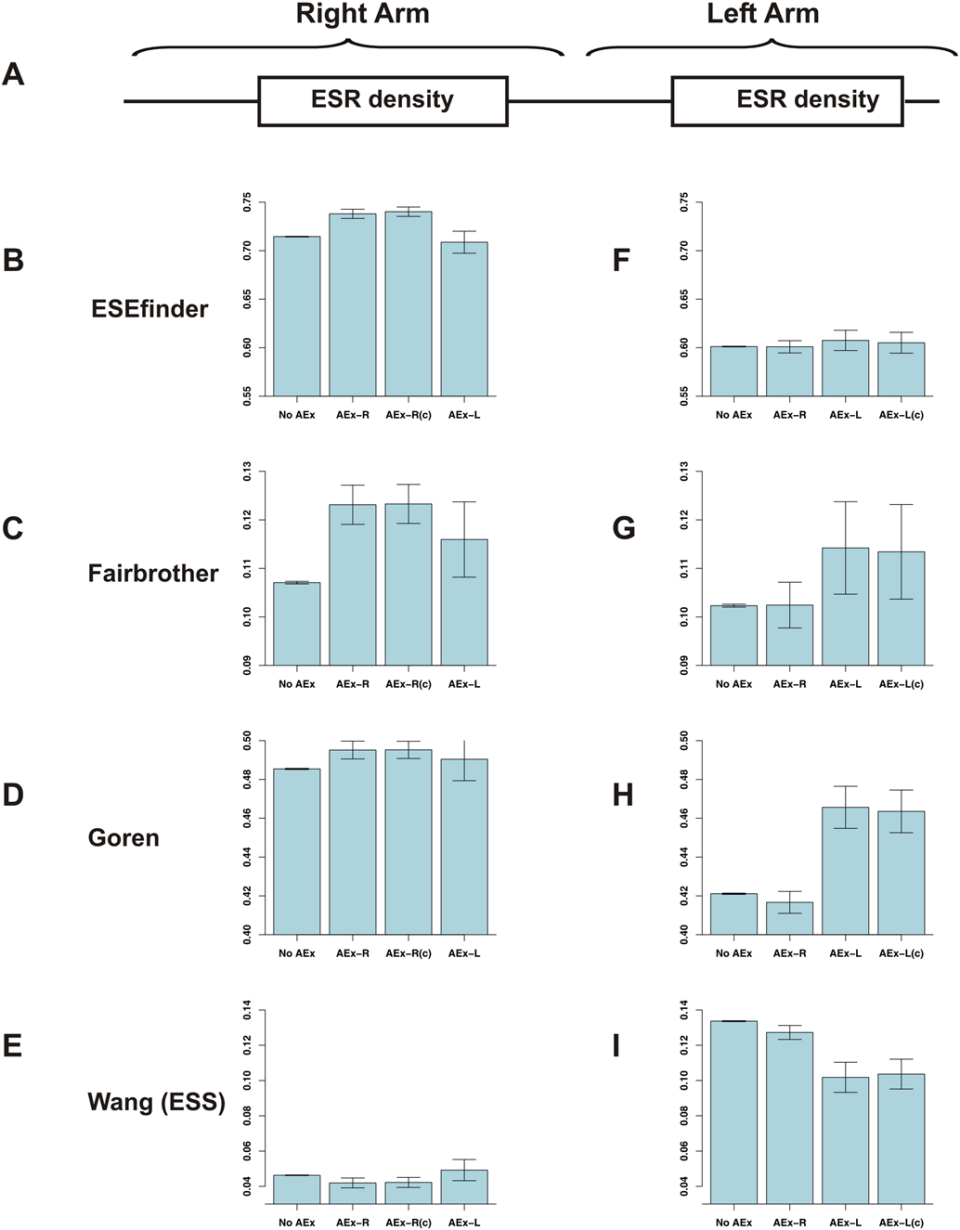
Analysis of ESR densities. (A) Diagram depicting the two *Alu* arms. (B–E) Mean ESR densities in the right arm *Alu* exon/non-exon, across the three core groups and the relevant control (AEx-R(c)). Values are presented for four groups of ESRs: the groups of SR-protein binding sites in ESEfinder [35], the dataset of ESEs of Fairbrother et al. [36], the ESR dataset of Goren et al. [37], and the ESSs of Wang et al. [38]. The ESR density is calculated as the total number of nucleotides that overlap with motifs from each dataset divided by the length of the exon. The error bars present the standard error. (F–I) ESR densities in the left arm across the different groups.

Based on both biologic and bioinformatic methodologies, datasets of exonic splicing enhancers (ESEs) and silencers (ESSs) have been compiled; these sequences are believed to increase or decrease, respectively, the spliceosome's ability to recognize exons. Indeed, exons were found to be enriched in ESRs with respect to pseudo-exons or exons [32]–[34]. Thus, our next step was to determine the densities of ESEs and ESSs in exons and non-exons. We made use of four datasets of exonic splicing regulators (ESRs): the groups of SR-protein binding sites in ESEfinder [35], the dataset of ESEs from Fairbrother et al. [36], the exonic splicing regulatory sequences compiled by Goren et al. that consists mostly of ESEs [37], and the ESS dataset compiled by Wang et al. [38]. For each exon (or non-exon) in the two *Alu* arms (Figure 4A) in the three core and two control datasets, we calculated the ESR density for the four groups of ESRs. The ESR density was calculated as the total number of nucleotides within an exon that overlap with motifs from a given dataset divided by the length of the exon.

We found that *Alu* exons showed a marked tendency for enrichment in ESEs and depletion in ESSs with respect to their non-exonizing counterparts. Right arm *Alu* exons had significantly higher densities of ESEfinder ESEs than their counterparts in the No AEx group (Figure 4B, p = 0.00007) and higher densities of ESEs from Fairbrother et al. (Figure 4C, p = 0.00009). Higher densities were also observed in terms of ESEs found in Goren et al. (Figure 4D), whereas slightly lower densities were observed for the ESSs of Wang et al (Figure 4E); However, the trends for the latter two datasets were not statistically significant. In the left arms, similar tendencies were observed: Exons originating from this arm were highly enriched in ESEs of Goren et al. (Figure 4H, p = 0.0001) and depleted in ESSs of Wang et al. (Figure 4I, p = 0.0003). They also tended to be enriched in ESEs of Fairbrother et al. (Figure 4G), although this was not significant (p = 0.12); and in this arm no differences were found in terms of ESEs of ESEfinder (Figure 4F, p = 0.72). To summarize, in all cases in which significant differences were observed, these differences reflect an increase in ESE densities in parallel with a decrease in ESS densities in exons relative to non-exons.

### Machine-Learning Mimicking of Spliceosomal Function

Since the splicing machinery is able to differentiate between exonizing and non-exonizing *Alus*, we were interested in discovering whether the features identified here can give rise to such precise classification. Toward these aims, we used Support Vector Machine (SVM) machine learning, which has shown excellent empirical performance in a wide range of applications in science, medicine, engineering, and bioinformatics [39]. We created two classifiers: One discriminating between non-exonizing *Alu*s and *Alu*s exonizing from the right arm and one discriminating between non-exonizing *Alu*s and *Alu*s exonizing from the left arm. Receiver-operator curves (ROC curves) were used to test performance. Briefly, ROC curves measure the tradeoff between sensitivity and specificity of a given classification. A perfect classification with 100% sensitivity and 100% specificity will yield an area under the curve (AUC) of 1, whereas a random classification will yield an AUC of 0.5 (see Materials and Methods for complete details of the SVM protocol used). 14 features were selected for the machine learning. These were divided into 5 clusters: 5′ss strength (1 feature: 5′ss score), 3′ss strength (1 feature: 3′ss score), secondary structure (5 features: z-scores for the stability of secondary structure of the entire *Alu* and of each of the two *Alu* arms, PU values of the 5′ss, and PU values of the 3′ss), exon-intron architecture (3 features: lengths of upstream intron, of *Alu* exon, and of downstream intron), and ESRs (4 features: density in terms of each of the 4 groups of ESRs).

**Figure 5:**
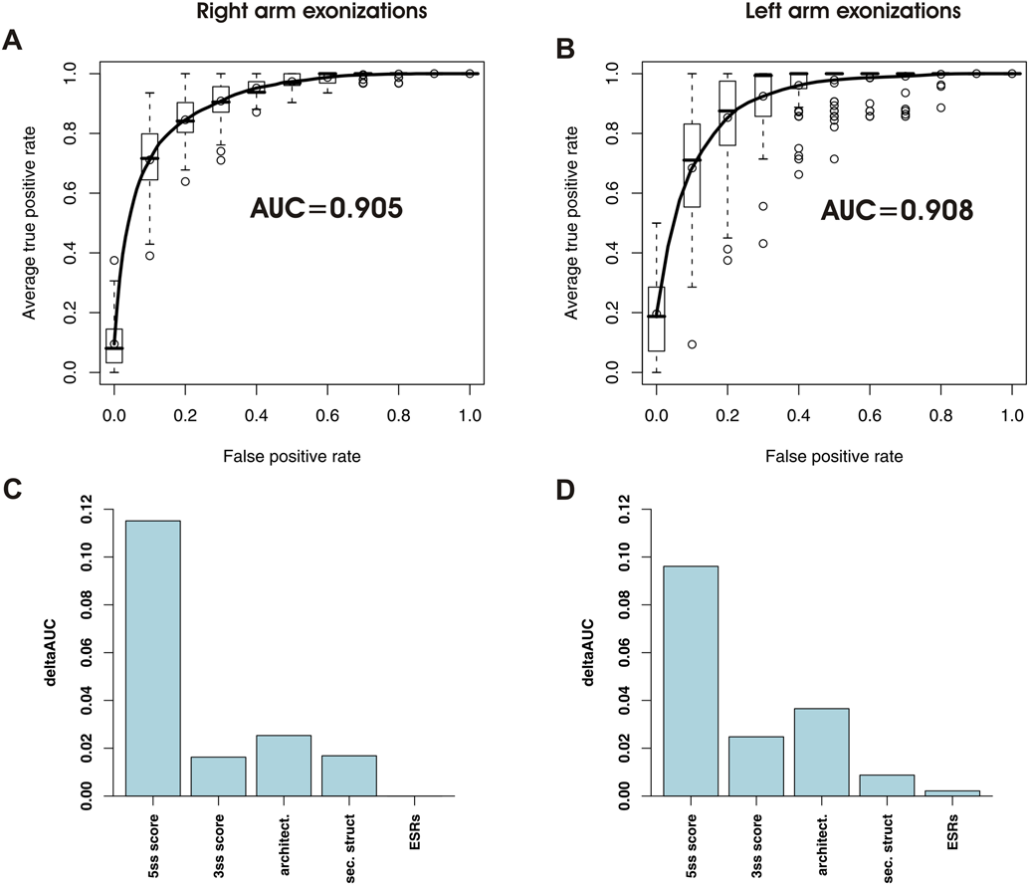
Results of SVM machine learning discriminating between true exons and non-exons in the *Alu* right arm. (A) Averaged receiver-operator curves (ROC) and mean area-under-the-curve (AUC) values for 10 cross-validation support vector machine (SVM) runs for classification between right arm exons in the AEx-R(c) group and in the No AEx group. (B) Analysis as in (A), but for left arm *Alus*. (C) ΔAUC values of each cluster of features indicating the contribution of each cluster to correct classification of non-exonizing *Alu*s and exonizing *Alu*s from the right arm. (D) Analysis as in C, but for the left arm.

Based on the above-described features, we were able to achieve a high degree of classification between exonizing and non-exonizing *Alu*s. Figure 5A presents the ROC curves and AUC values for the classification between *Alu*s exonizing from the right arm and non-exonizing *Alu*s and Figure 5B presents these values for the classification between the *Alu*s exonizing from the left arm and the non-exonizing ones. The AUC values of ∼0.91, demonstrate that our features achieve a high degree of accuracy in discriminating between true exons and non-exons, thus mimicking the role of the splicing machinery.

If selection of an *Alu* exon is indeed determined by this set of features, then this same set of features may well also determine the inclusion level of an *Alu* exon. A “strong” set of features will lead to a high selection rate by the spliceosome, and hence to high inclusion levels, whereas “weaker” features may lead to a more reduced selection rate by the spliceosome and to lower inclusion levels. Indeed, we found a positive, highly significant correlation between probabilities of exonization based on the SVM model and between inclusion levels of exons based on EST data in the case of right arm *Alu* exons (Pearson, r = 0.28, p = 6.35e−07). For the sake of comparison, the correlation between 5′ss scores and inclusion levels is considerably lower and less significant (r = 0.15, p = 0.007). Thus, although the computational model was explicitly trained on the basis of a dichotomous input (*Alus* were labeled either as exonizing or as non-exonizing), the model managed to capture the more stochastic nature of the spliceosomal recognition of exons. A positive correlation existed in the left arm as well, but this correlation was not significant presumably due to the fewer number of *Alus* in the AEx-L dataset.

Although our model was trained on *Alus*, and specifically on comparing non-exonizing *Alus* to mostly alternatively recognized *Alus*, we reasoned that the same set of features which make the difference between a non-recognized and an alternatively-recognized *Alu* exon might also make the difference between an alternatively recognized exon and a constitutively recognized one. We therefore applied the SVM model to datasets of constitutive and cassette exons. For this purpose, we generated a dataset of 55,037 constitutive and 3,040 cassette exons based on EST-data (see Materials and Methods). For each of these exons, we first extracted all above-described features, and then applied the SVM model to them. Our model classified constitutive and alternative exons as different in a highly statistically significant manner. The mean probability of undergoing exonization, provided by the logistic regression transformed SVM model, was 73% for the constitutive exons, but only 60% for the alternative ones (Mann-Whitney, p<2.2e−16). In addition, 82% of the constitutive exons were classified as “exonizing”, in comparison to only 63% of the alternative exons. These results demonstrate that the features learned by the SVM model are relevant for exonization in general, and control not only the shift of non-exons to alternative ones, but also of alternative exons to constitutive ones.

Finally, we were interested in assessing the importance of different features in allowing correct discrimination between exonizing and non-exonizing elements. For this purpose, we used ΔAUC to measure the contribution of each feature cluster. This measure compares the performance of the classification with and without each cluster of features, with greater differences indicating greater contribution of a given cluster of features to precise classification. The feature with the highest contribution, both in the right arm (Figure 5C) and in the left arm (Figure 5D), was the strength of the 5′ss, in concordance with previous bioinformatic findings [26]. However, much information is included in the other features as well. The second most important feature both in the left and in the right arm was exon-intron architecture. Secondary structure and the 3′ss had a comparable contribution in the right and left arm. Despite the differences in terms of ESR densities between the different datasets, this feature cluster had a negligent contribution to classification in the right arm, and a slightly higher one in the left arm. Using a mutual information based metric to measure the contribution of the different features, yielded similar, consistent results (see Text S1).

## Discussion

In this study, we sought to determine how the splicing machinery distinguishes true exons from non-exons. *Alu* exonization provided a powerful model for approaching this question. Exonizing *Alu*s have retained high sequence similarity to their non-exonizing counterparts but are perceived differently by the splicing machinery. Past studies have emphasized mainly the splice sites, but our results indicate the importance additional features that lead to exonization. These features, which include splicing signals (splice sites and ESRs), exon-intron architecture, and secondary structural features, achieved a high degree of classification between true *Alu* exons and non-exons, demonstrating the biological relevance of these layers in determining and controlling exonization events.

Perhaps the most interesting result to emerge from this study is that secondary structure is critical for exon recognition. It has been assumed that pre-RNA is coated *in vivo* by proteins [10] and that these RNA-protein interactions either prevent pre-mRNAs from folding into stable secondary structures [40] or provide pre-mRNAs with a limited time span for folding [41]. However, an increasing number of studies are finding that secondary structure plays a crucial role in the regulation of splicing. Secondary structures involving entire exons (e.g., [5]–[7]), the splice sites only (e.g., [8],[11],[12]), or specific regulatory elements [42],[43] were shown to be involved in the regulation of alternative splicing. Hiller et al. [14] recently found that regulatory elements within their natural pre-mRNA context were significantly more single stranded than controls. Our current study puts these findings into a broad context, and provides bioinformatic evidence for the notion that the structural context of splicing motifs is part of the splicing code. Such a structure, as we have shown, is detrimental for exonization in general, and specifically if it overlaps the 5′ss.

**Figure 6:**
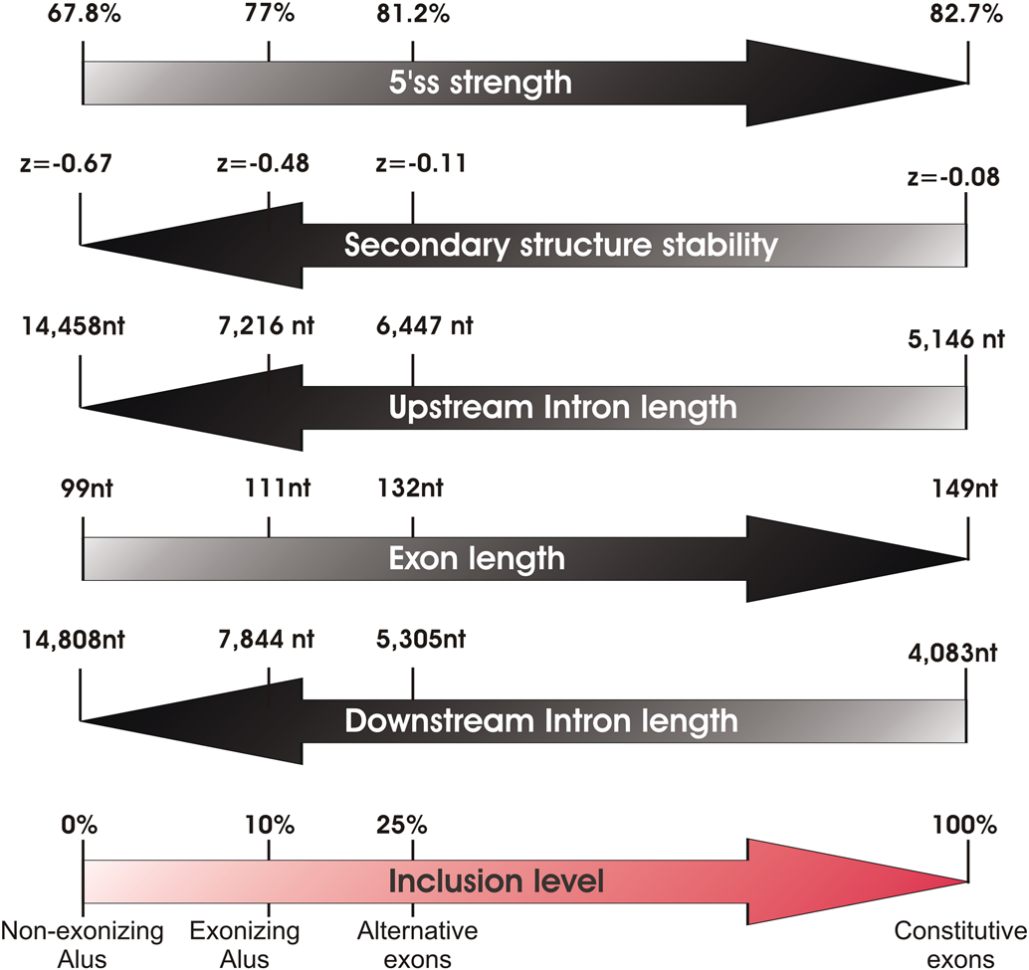
Diagram depicting gradual changes in different factors correlating with exonization levels. Inclusion levels are shown to gradually increase from non-exonizing *Alus*, to exonizing *Alus*, to alternative exons, to constitutive exons. This increase correlates positively with exon lengths and with 5′ss strength, and negatively with lengths of flanking introns and predicted secondary structure stability of the exon.

Several intriguing observations can be made when merging our results based on the exonizing and non-exonizing *Alu*s with those of the alternative and constitutive datasets. In terms of inclusion level, these four groups form a continuum, with non-exonizing *Alus* having a 0% inclusion level, exonizing *Alus* having a mean inclusion level of 10%, cassette exons having a mean inclusion level of 25%, and constitutive exons being included in 100% of the cases. Gradual changes when moving from non-exonizing *Alus*, to exonizing *Alus*, to alternative exons, to constitutive ones are observed in several additional features: The strength of the 5′ss gradually increases from non-exonizing *Alus* to constitutive exons, the strength of the secondary structure gradually decreases, lengths of the upstream and downstream introns gradually decrease while length of the exons gradually increase (see Figure 6 for detailed values). These gradual changes are all coherent in biological terms: Stronger 5′ splice sites allow higher affinity of binding between the spliceosomal snRNAs and the 5′ss, and have well documented effects in increasing exon selection [28],[44]; stronger secondary structure can sequester binding sites of spliceosomal components; And it has been previously shown that longer flanking introns profoundly increase the likelihood that an exon is alternatively spliced [4], and that alternative exons tend to be shorter than their constitutive counterparts (reviewed by [16]), presumably due to spliceosomal constraints. In addition, our finding that selective constraints are simultaneously applied both on the lengths of the exons and of their flanking introns suggests that the exon and its flanking introns are recognized, to some extent, as a unit. This challenges the more traditional exon-definition and intron-definition models [3],[45], according to which either the exon, or its flanking introns, but not both, are recognized by the splicing machinery.

Notably, in our search for features differentiating between exonizing and non-exonizing *Alus*, we focused only on features which can potentially be mechanistically employed by the splicing machinery to differentiate between exons and introns. For this reason, we did not use phylogenetic conservation, nor the age of the *Alu* exons, nor the location of the exonization event (CDS vs. UTR) as features. Although these features are informative as well (see Text S1, and [32]), and thus may potentially boost the performance of our classifier, these cannot be directly sensed by the spliceosome. Rather, these elements reflect the evolutionary pressures to which an exonizing *Alu* element is subjected.

In our study we found that introns flanking exonizing *Alus* are dramatically shorter than the introns flanking their non-exonizing counterparts. These results appear to contradict recent results [46] according to which there is a tendency for new exons to form within longer introns. However, two points must be borne in mind in this context: First, the introns flanking exonizing *Alus* are longer than average introns, and thus our results are consistent with the above study in that exonizations occur in longer introns. Second, our findings may reflect an upper bound in terms of intron length within which exonization optimally occurs, and introns longer than a certain threshold may cease to be good candidates for exonization.

Our results indicate that the *Alu*-trained model could be applied to a more general context of alternative and constitutive exons, where it yielded coherent results. This does not, however, imply that all findings made in the context of *Alus* can be directly extrapolated to exons in general. For *Alu* sequences, we found the 5′ss to be the most informative feature for correctly predicting exonization events, in agreement with previous findings [26],[28]. We found, however, that the 3′ss, which was also found to play a major role in exonization [24], is less critical. This finding may not necessarily hold for all exons. The relatively low contribution of the 3′ss to *Alu* exonization may reflect the general tendency of *Alu*s to have relatively strong splice signals at their 3′ end, regardless of whether they undergo exonization or not. This is since the poly-T track, present in all *Alu*s in the antisense orientation, serves as a strong polypyrimidine tract [24],[47]. On the other hand, our results regarding the importance of ESRs are consistent with several previous studies that have found exons to be enriched in ESRs with respect to pseudo-exons, more poorly recognized exons, and introns [32]–[34]. Thus, while the importance of different features may vary from one exon to another, our results provide a general understanding of the features impacting on exon recognition.

It is noteworthy, that the majority of *Alu* exonization events in our two exonizing datasets presumably reflect either errors of the splicing machinery or newly born exons, which presumably do not give rise to functional proteins (see also [48]). This is indicated by the low inclusion level of the *Alu* exons, averaging 13% and 10% in the AEx-R and AEx-L groups, respectively. In addition, the symmetry of the *Alu* exons (i.e., divisibility-by-three), at least in the AEx-R dataset, is very low: Only 23% of the exons are symmetric (in the AEx-L dataset 55% of the *Alu*s are symmetric). Thus, the majority of *Alu*s in this dataset insert a frame-shift mutation. These numbers contrast with the 73% symmetry found in alternative events conserved between human and mouse [49]. However, since our objective in this research was to understand the requirements of the spliceosome, the potential function of the transcript is irrelevant. Moreover, newly born alternatively spliced *Alu* exons are the raw materials for future evolution: Given the right conditions and time, further mutations might generate a functional reading frame.

The features identified here provided good, but not perfect, classification using machine learning. A number of factors underlie the non-perfect classification: For example, EST data is very noisy and far from providing a comprehensive coverage of all genes in all tissues [50]. Therefore, many *Alu*s categorized as non-exonizing may, in fact, undergo exonization in certain tissues. Moreover, the features uncovered here may well not be exhaustive. Finally, as suggested by the correlation between the strength of predictions and the inclusion level, the alternative splicing pattern of the *Alu* exons may imply that the spliceosome itself does not perfectly recognize the exons. In this sense, the non-perfect classification of the machine learning model may reflect, to some extent, the non-perfect selection of the splicing machinery, giving rise to alternative events.

## Materials and Methods

### Compilation of Datasets

We compiled a dataset of intronic *Alu*s in the antisense orientation that do not undergo exonization and datasets of *Alu*s that are exonized in their right arms and left arms. We used a similar, but improved, procedure to the one described in [28]. We retrieved all human intronic *Alu*s in the antisense orientation by querying the TranspoGene database [51]. Using the needle application [52], we next performed pairwise, global alignments between the *Alu* sequences and the *Alu-Jo* consensus sequence which was downloaded from RepBase [53] (http://www.girinst.org/). Since we desired only *Alu*s sharing a ‘reasonable’ degree of similarity which would ensure a common basis for comparison, we next filtered out all *Alu*s with over 40 indels relative to the *Alu* consensus sequence; this cutoff was set empirically. Finally, we filtered out all redundant entries based on overlapping genomic coordinates. We next identified all cases in which EST evidence (based on the TranspoGene query) supported exonization from the right arms and from the left arms. These *Alu*s formed the initial AEx-R and AEx-L datasets. To form the No AEx group, we began with all *Alu*s lacking any evidence of exonization and retained only those *Alu*s overlapped by ≥20 ESTs, based on the hg17 ‘Spliced ESTs’ table downloaded from the UCSC website (http://genome.ucsc.edu/).

### Finding Optimal Potential Splice Sites

To identify optimal exon boundaries (i.e. flanking 3′ and 5′ splice sites) in the left arms of the AEx-R dataset, in the right arm of the AEx-L dataset, and in both arms of the No AEx dataset, we first characterized the position windows in which 5′ and 3′ splice sites tended to be located, in the right and left arms of sequences in the AEx-R and AEx-L datasets, respectively, as in [28]. The 3′ss was defined as the 15-nucleotide (nt) sequence covering the 14 last intronic nucleotides and the first exonic nucleotide and the 5′ss was defined as a 9-nt sequence covering the 3 terminal exonic nucleotides and the first 6 intronic nucleotides. We found that 97% of the 3′ss in the right arm of the AEx-R dataset were located upstream of position 58 (relative to the consensus) and that >98% of the 5′ss in the right arm of the AEx-R were located between positions 105 and 181. Similarly, >95% of the 3′ss were located between position 181 and 204 and all 5′ splice sites in the left arm of the AEx-L group were downstream of position 249. We next searched for the highest scoring splicing signals within the relevant positional windows of the left arm of the AEx-R group, the right arm of the AEx-L group, and both arms of the No AEx group. *Alu*s lacking a minimal potential splice site in either arm, defined as an ‘AG’ for the 3′ss and a GT/C for the 5′ss, were filtered out. Splice site scores were determined by first calculating log-odd scores based on position specific scoring matrices (PSSMs) of the relevant splicing signal and subsequently rescaling them to lie between 0 and 100, as described in [54]. The 5′ss PSSM, spanning 3 exonic and 6 exonic positions, was derived from the Analyzer Splice Tool webserver (http://ast.bioinfo.tau.ac.il/SpliceSiteFrame.htm), and the 3′ss PSSM, spanning 14 intronic and 1 exonic position, was derived from [55]. The two control datasets, AEx-R(c) and AEx-L(c) were created based on the same set of *Alus* as the AEx-R and AEx-L groups, respectively, but by defining exon borders in both arms based on the computational prediction rather than on EST evidence.

### Z-Scores of Secondary Structure Strength

The predicted free energy of the ensemble of all secondary structures of a sequence was obtained via the RNAfold application in the Vienna RNA Package [29],[56]. However, these measures are highly sensitive to sequence length and to dinucleotide composition [57]. To overcome these biases, we used DiShuffle [58] to generate 50 random sequences from each original sequence sharing its length and dinucleotide composition. The Z-scores were calculated as the difference between the free energy of the original sequence and the mean partition function of the 50 randomized sequences, divided by the standard deviation of the partition functions of the randomized sequences.

### Measurement of Single Strandedness (PU Values)

PU values represent the probability that all bases in a motif are unpaired (denoted as probability unpaired or PU value) [14]. These motifs were calculated as in [31]. Briefly, the PU value for the region *a* to *b* in an mRNA sequence is defined as:
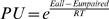
where *E_all_* is the free energy of the ensemble of all structures, *E_unpaired_* is the free energy of the ensemble of all structures that have the complete region *a* to *b* unpaired, *R* is the universal gas constant, and *T* is the folding temperature. *E_all_* and *E_unpaired_* were computed using the partition function version of RNAfold [29]. For *E_unpaired_*, we assured that the region *a* to *b* was unpaired by applying additional constraints (RNAfold parameter-C).

To reduce the dependency on a single fixed context length, we considered all symmetrical context lengths from 11 up to 31 nt upstream and downstream of the splicing motif in increments of 5, similar to [14]. Thus, for a 5′ss motif of length 9 nt, we considered sequences with a total length of 31 nt (for context length of 11, 2×11+9 = 31), 41 nt (for context length 16), 51 nt, 61 nt, and 71 nt (for context length of 31). We computed the PU value of the splicing motif for each of these context lengths and averaged them.

### Determining Lengths of Flanking Introns

To determine the length of the introns flanking the *Alu* exons/non-exons, we downloaded the UCSC hg18 Known Genes track, creating a separate record for each exon. We used the LiftOver application (available in http://hgdownload.cse.ucsc.edu/downloads.html) to convert the *Alu* start and end coordinates from hg17 (used by TranspoGene) to hg18. For each *Alu* in each of the datasets, we identified the most proximal exon upstream and downstream of the *Alu*, based on which we calculated lengths of the introns flanking the *Alu* elements.

### SVM Machine Learning

Our aim was to build two classifiers discriminating between *Alu*s undergoing and not undergoing exonization: one between the *Alu*s undergoing exonization in the right arm and the non-exonizing ones and one between *Alu*s undergoing exonization in the left arm and the non-exonizing ones. Since we had two datasets representative of *Alu*s exonizing from the right (AEx-R and AEx-R(p)) and two from the left (AEx-L and AEx-L(p)), in practice we built four classifiers, with each classifier distinguishing between the No AEx group and one of the above four groups.

The machine learning approach we decided to use was support vector machine (SVM). We made use of the e1071 package [59], which provides an interface to LIBSVM [60] in R statistical package [61]. All variables were normalized prior to the machine learning process, to zero mean and unit variance. The variables of intron length and PU values were first log-transformed, as well.

The difference in several orders of magnitude between the size of the datasets of *Alu*s undergoing and not undergoing exonization causes the instance of the former to ‘drown’ within the latter. In our machine learning, we therefore maintained a 3∶1 ratio between non-exonizing and exonizing *Alu*s by randomly selecting three *Alu*s from within the non-exonizing dataset for each *Alu* in the exonizing dataset.

SVM training involves fixing several hyper-parameters, which have a crucial effect on the performance of the trained classifier [39]. To identify an optimal hyper-parameter set, we used 10-fold cross-validation on the training set and performed a grid search with linear, polynomial, and Gaussian kernels and with a range of cost and gamma values. For this purpose, we used the tune() function in the e1071 package. We found that in the majority of cases the SVM performed best with a linear kernel and a cost factor of 1.

Evaluation of the SVM prediction was achieved by implementing a 10-fold stratified cross-validation procedure (i.e., in each run maintaining the 3∶1 ratio between the training and test sets) using the area under the ROC curve (AUC) as a global performance measure. We performed 10 cross-validation runs (in each such run using a different set of randomly selected non-exonizing *Alu*s) and the 100 ROC curves from these runs were averaged and displayed in Figure 6 via the ROCR package [62]. The mean AUC in these runs was calculated as well, as an overall performance measure. Logistic regression fitted to the decision values of the SVM classifier was applied using the probability = TRUE option in the svm() function.

### Statistical Analysis

Unless explicitly stated otherwise, the hypothesis that a factor distributed equally across different groups was tested using the non-parametric Kruskal-Wallis one way analysis of variance test. As post-hoc tests, we then performed Mann-Whitney tests between each pair of groups. The results for these tests are all presented in Text S1.

### Compilation of Constitutive and Alternative Datasets

Coordinates of human (hg18) exons based on the Refseq track and coordinates of spliced EST alignments were downloaded from the UCSC genome browser (http://genome.ucsc.edu/). For an EST to support exon inclusion, we demanded that the exon be either fully included in the alignment of the EST sequence, or that at least 50 nt of the exon and either of its two splicing signals form part of the alignment. Alignment gaps of less than 8 nt were ignored, as in the UCSC visualization defaults. An EST was defined as supporting exon skipping if no alignment between them was observed, and if the EST was defined as supporting the two flanking exons. For the constitutive exons, we selected all exons whose inclusion was supported by at least 20 ESTs and lacking any ESTs supporting exon skipping, whereas for the alternative dataset we selected all exons with at least 5 ESTs supporting inclusion and 5 ESTs supporting skipping. As a final step, the features described in the manuscript were extracted for each of the exons in the two datasets, with the exception of secondary structure of the opposite *Alu* arm and within the entire *Alu*, since these features cannot be applied to exons in general.

## Supporting Information

Text S1 Supplementary Methods, Tables, and Figures
(0.15 MB PDF)
