# Supplementary material for: Alu Exonization Events Reveal Features Required for Precise Recognition of Exons by the Splicing Machinery
Source: PLoS Comput Biol. 2009 Mar 6;5(3):e1000300. doi: 10.1371/journal.pcbi.1000300 (PMC2639721; doi:10.1371/journal.pcbi.1000300)
Supplement: Text S1 — Supplementary Methods, Tables, and Figures (0.15 MB PDF) [file pcbi.1000300.s001.pdf]

Alu exonization events illuminate the features required for exon selection / Schwartz et al / Text S1

Supplementary Methods

Calculation of PU values

A possible bias in the analysis presented in Figure 2, panels E-G, concerning depletion of local secondary structures overlapping the 5'ss, might be that the decreased secondary structure of the entire exonizing arm (as shown in Figure 2, panels B-D) may result in a decreased structure throughout the entire *Alu* exon. Given such a scenario, the observed single-strandedness of the 5'ss may not be specific to the 5'ss region; rather, any randomly chosen site throughout the exonizing arm might be relatively less structured. To address this, we arbitrarily selected nine equally distanced sites within the right and the left *Alu* arms, beginning at position 40 and ending at position 280 sampling one site every 30 nt. We calculated the PU value of the 9-mer (the length of the 5'ss) at each site within each of the three core datasets. For five of the nine sites, no significant differences in PU values (at a level of $p<0.05$) were found between the groups. In the four remaining sites, although differences between datasets were significant, the PU values were not consistently higher in the exonizing dataset. The fact that consistently higher, statistically significant, PU values were found specifically for the recognized 5'ss but not for the randomly selected position therefore implies potential biological importance.

Ranking of features

Mutual information is a quantity that measures the mutual dependence of two variables, and is calculated as:

$$I(X;Y) = \sum_{y \in Y} \sum_{x \in X} p(x,y) \log\left(\frac{p(x,y)}{p_1(x)\, p_2(y)}\right)$$

where $p(x,y)$ is the joint probability distribution function of X and Y, and $p_1(x)$ and $p_2(y)$ are the marginal probability distribution functions of X and Y respectively. For calculating this value we made use of the bioDist()

package [1] in R, which first discretizes each variable by binning them into 10 bins. The mutual information of each variable was next normalized (divided) by the entropy of the binary 'group' variable which indicates whether an Alu exonizes from a given arm or not. The entropy of this variable, H(x), was calculated as:

$$H(x) = -\sum_{i \in \{0,1\}} p(x_i) \log(p(x_i))$$

where $p(x_i)$ is, in turn, the probability that an *Alu* does and does not undergo exonization. The final value was multiplied by 100, to yield the percentage of information. This measure has previously been termed coefficient of constraint [2] and uncertainty coefficient [3].

The mutual information of each of the variables with respect to the target variable (i.e. a boolean variable indicating whether an *Alu* exonizes or not) is presented in Supplementary Figure 1A, for classification between right arm exonization and non-exonizing *Alus*, and in Supplementary Figure 1B for the left arm exonizations.

Analysis of *Alus* by inclusion levels

It has recently been shown that older Alu exons are characterized by stronger signals and higher inclusion levels than younger ones [4]. We were thus interested in determining whether the different features identified in this study are stronger in Alu exons characterized by higher inclusion levels. To assess the impact of inclusion level, we divided all 313 Alus exonizing from the right arm into two groups of low and high inclusion levels, using an inclusion level threshold of 20% to divide the groups. This left 263 and 50 Alu exons in the LOW and HIGH inclusion groups. For each of the features described in the manuscript, we next used t-tests to determine whether they significantly differed between the two groups. Two features were found to be significantly different in the two groups: the 5'ss score (mean in LOW – 76.21, mean in HIGH – 80.54, P-value - 0.003), and right arm secondary structure (mean Z-score in LOW - -0.51, mean in HIGH - -0.27, P-value – 0.001). This analysis again underscores the importance of

secondary structure, which in this case was even more significant than that of the 5'ss. Full results for this analysis can be found in Supplementary Table 2. Repeating this analysis in the left arm did not yield significant results, which is at least partially to be attributed to the much lower number of *Alus* in this dataset.

Analysis of *Alus* by location of exonization

We have previously reported a tendency for exonization events of transposable elements to occur within the UTRs [5]. Analyzing this in our datasets, we find that that of the 313 Alus exonizing from the right arm, 109 occur in the 5' UTR, 198 in the CDS, and only 6 in the 3' UTR. We were next interested in determining whether Alu exonizations from the UTR are characterized by different properties than CDS exonizations. To this end, we retained only *Alus* exonizing within either the 5'UTR and CDS, and compared the different features identified in the manuscript. Significant differences were found in terms of length of flanking introns, secondary structure, and two groups of ESEs, whereas all the remaining features did not differ significantly. See Supplementary Table 2 for full results.

Supplementary Tables

	Variable	p value multiple compar.	p value NO vs RIGHT	p value NO vs LEFT	p value RIGHT vs LEFT
Splicing Signals	Right 3'ss score	1.93E-04	4.81E-05	0.44721	0.0365557
	Right 5'ss score	0	0	0.74765	1.24E-15
	Left 3'ss score	1.20E-05	0.97713	1.92E-06	5.11E-05
	Left 5'ss score	0	0.19342	0	8.88E-16
Secondary Structure	Sec. Structure Entire Alu	1.78E-11	9.86E-12	0.07395	0.1524639
	Sec. Structure Right Arm	1.11E-16	2.00E-15	0.00177	0.4502867
	Sec. Structure Left Arm	1.01E-05	0.05517	1.08E-05	0.002153
	PU right arm 5'ss	0.454669	0.2233	0.75814	0.7705114
	PU left arm 5'ss	0.007229	0.27216	0.00324	0.0278523
	PU position 156	0.006698	0.00212	0.45607	0.0745562
	PU position 176	0.002235	0.00224	NA	NA
	PU position 291	0.09565	0.76786	0.03196	0.0390229
Intron-Exon Architecture	Length Right Arm exon	0	0	0.15144	0.002001
	Length Left Arm exon	0.326815	0.23571	0.36551	0.0628657
	Length Left Arm exon (anova, t-test)	3.50E-04	0.31636	1.14E-04	3.58E-05
	Upstream Intron Length	2.54E-14	1.38E-13	0.00478	0.4767794
	Downstream Intron Length	7.11E-15	6.15E-12	2.22E-05	0.175689
Exonic Splicing Regulators	Right density ESEfinder	3.31E-04	7.38E-05	0.58094	0.0170781
	Right density Goren	0.187137	0.09669	0.43939	0.9653458
	Right density Fairbrother	2.50E-04	9.22E-05	0.2518	0.4447262
	Right density Wang (ESS)	0.385217	0.19155	0.65569	0.2782349
	Left density ESEfinder	0.922704	0.84864	0.72467	0.6672109
	Left density Goren	4.15E-04	0.42282	1.12E-04	1.41E-04
	Left density Fairbrother	0.308756	0.86833	0.12751	0.1637287
	Left density Wang (ESS)	7.32E-04	0.24362	2.97E-04	0.0031481

Supplementary Table 1. Statistical significance of features across the three core datasets. For each feature, four tests were performed: a first general Kruskal-Wallis one way analysis of variance test (or ANOVA if explicitly stated), to assess whether the level distributed differently across the three core datasets, followed by three Mann-Whitney tests (or t-tests) between each pair of datasets, to identify which datasets differed from others. P-values beneath 0.05 are highlighted in yellow.

	Low inclusion	High Inclusion	P value		5' UTR	CDS	P value
5'ss score	76.21	80.54	0.003		78.14	76.09	0.094
3'ss score	87.10	87.67	0.526		87.26	87.15	0.852
exon length	110.44	114.88	0.062		113.23	109.87	0.098
log(up intron length)	7.91	7.66	0.332		8.26	7.67	0.002
log(dn intron length)	7.95	7.92	0.913		8.28	7.77	0.003
Sec. Str. Z-score	-0.51	-0.28	0.001		-0.36	-0.53	0.004
PU 3'ss	-2.89	-2.93	0.917		-2.89	-2.94	0.867
PU 5'ss	-6.78	-6.62	0.697		-6.52	-6.82	0.335
ESEfinder	0.73	0.76	0.051		0.75	0.73	0.037
Goren	0.50	0.48	0.273		0.49	0.50	0.215
Fairbrother	0.12	0.13	0.211		0.11	0.13	0.012
Wang	0.04	0.04	0.272		0.04	0.04	0.187

Supplementary Table 2: This table presents results for two analyses performed on the 313 *Alu* exonizations in the right arm. In the first, exons were divided into two groups based on their inclusion levels (above and below 20%), and in the second based on location (5’ UTR vs. introns). T-tests were performed to compare each feature in each of the two groups. Significant p-values (P<0.05) are highlighted in yellow.

Supplementary Figures

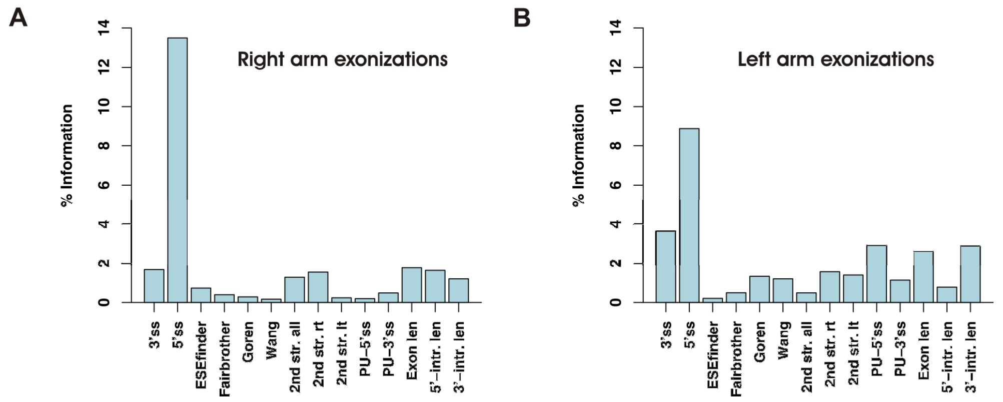

Supplementary Figure 1: Degree of informativeness of different features, based on a mutual-information derived metric indicating to what extent each of the features is informative in terms of predicting whether an *Alu* will undergo exonization or not. Higher values indicate that a feature is more informative in terms of predicting whether the *Alu* will exonize from a given arm. Panel **A** presents the relative information of each feature in terms of classification between exons originating from the right arm and the non-exonizing ones, and Panel **B** presents these values for the classification between left arm exonizing *Alus* and non-exonizing ones. Abbreviations: 3’ss and 5’ss – splice site scores; ESEfinder, Fairbrother, Goren, Wang – densities in terms of ESRs; 2nd str. all – secondary structure Z-scores of entire *Alu*; 2nd str. rt, 2nd str. lt – secondary structure Z-scores of right and left arms, respectively; PU-5’ss, PU-3’ss – PU values for the 3’ss and 5’ss, respectively; Exon len – length of exon; 5’-intr. len, 3’-intr. len – length of introns downstream and upstream of the exon, respectively.

References

1. Ding B, Gentleman R, Carey V bioDist: Different distance measures.
2. Coombs CH, Dawes RM, Tversky A (1970) Mathematical Psychology: An Elementary Introduction. Prentice-Hall, Englewood Cliffs, NJ.
3. Press WH, Flannery BP, Teukolsky SA, Vetterling WT (1988) Numerical Recipes in C: The Art of Scientific Computing. Cambridge University Press, Cambridge: 634.
4. Corvelo A, Eyras E (2008) Exon creation and establishment in human genes. Genome Biol 9: R141.
5. Sela N, Mersch B, Gal-Mark N, Lev-Maor G, Hotz-Wagenblatt A, et al. (2007) Comparative analysis of transposed element insertion within human and mouse genomes reveals Alu's unique role in shaping the human transcriptome. Genome Biol 8: R127.
